# Activation of PPAR-β/δ Attenuates Brain Injury by Suppressing Inflammation and Apoptosis in a Collagenase-Induced Intracerebral Hemorrhage Mouse Model

**DOI:** 10.1007/s11064-020-02956-w

**Published:** 2020-01-14

**Authors:** Xiangming Tang, Kunning Yan, Yingge Wang, Yaping Wang, Hongmei Chen, Jiang Xu, Yaoyao Lu, Xiaohong Wang, Jingyan Liang, Xinjiang Zhang

**Affiliations:** 1 Institute of Translational Medicine, Medical College, Yangzhou University, Yangzhou, 225001 China; 2 Department of Neurology, Affiliated Hospital of Yangzhou University, Yangzhou, 225001 China; 3 Department of Electrocardiogram, Affiliated WuTaiShan Hospital of Medical College of Yangzhou University, Yangzhou Mental Health Centre, Yangzhou, 225000 China; 4 School of Nursing, Yangzhou University, Yangzhou, 225009 China; 5 General Hospital of Xuzhou Mining Group, Xuzhou, 221006 China; 6 Jiangsu Key Laboratory of Integrated Traditional Chinese and Western Medicine for Prevention and Treatment of Senile Diseases, Yangzhou University, Yangzhou, China; 7 Jiangsu Co-Innovation Center for Prevention and Control of Important Animal Infectious Disease and Zoonoses, Yangzhou, China

**Keywords:** Intracerebral hemorrhage, PPAR-β/δ, GW0742, Neuroprotection, Inflammation, Apoptosis

## Abstract

Brain injury has been proposed as the major cause of the poor outcomes associated with intracerebral hemorrhage (ICH). Emerging evidence indicates that the nuclear receptor, peroxisome proliferator-activated receptor β/δ (PPAR-β/δ), plays a crucial role in the pathological process of central nervous impairment. The present study was undertaken to evaluate the protective effects of PPAR-β/δ activation using a selective PPAR-β/δ agonist, GW0742, against brain injury after ICH in a mouse model. ICH was induced by intravenous injection of collagenase into the right caudate putamen. To examine the protective effect of PPAR-β/δ activation against ICH-induced brain injury, mice were either intraperitoneally injected with GW0742 (3 mg/kg, body weight) or saline (control group) 30 min before inducing ICH. Behavioral dysfunction was evaluated 24 and 72 h after injury. Then, all mice were killed to assess hematoma volume, brain water content, and blood–brain barrier (BBB) permeability. TUNEL and Nissl staining were performed to quantify the brain injury. The expression of PPAR-β/δ, interleukin (IL)-1β, tumor necrosis factor (TNF)-α, Bcl-2-related X-protein (Bax), and B-cell lymphoma 2 (Bcl-2) in the perihematomal area was examined by immunohistochemistry and western blotting analysis. Mice treated with GW0742 showed significantly less severe behavioral deficits compared to the control group, accompanied by increased expression of PPAR-β/δ and Bcl-2, and increased expression of IL-1β, TNF-α, and Bax decreased simultaneously in the GW0742-treated group. Furthermore, the GW0742-pretreated group showed significantly less brain edema and BBB leakage. Neuronal loss was attenuated, and the number of apoptotic neuronal cells in perihematomal tissues reduced, in the GW0742-pretreated group compared to the control group. However, the hematoma volume did not decrease significantly on day 3 after ICH. These results suggest that the activation of PPAR-β/δ exerts a neuroprotective effect on ICH-induced brain injury, possibly through anti-inflammatory and anti-apoptotic pathways.

## Introduction

Intracerebral hemorrhage (ICH) is a serious global health problem, accounting for 10–15% of all strokes worldwide [1]. The mortality rate of patients with ICH is about 30% within 1 month of disease onset, and survivors are left with varying degrees of disability and neurological complications [2]. Although advances have been made in the treatment of patients with ICH in recent years, few treatments have shown obvious clinical benefits. Therefore, there is a need to devise new therapeutic strategies for ICH.

ICH is usually caused by rupture of a blood vessel in the brain parenchyma, which leads to mechanical injury due to hematoma and toxicity from certain blood components [3]. ICH-induced pathophysiological changes include neuroinflammation, excitotoxicity, and neural cell apoptosis. The inflammatory response is a significant element of the pathological process of ICH, resulting in brain injury and neurobehavioral damage [4]. The most important features of the inflammatory response are the activation of microglia, infiltration of leukocytes, and release of a large number of cytokines and chemokines [5]. Numerous studies have shown that the inflammatory reaction plays a vital role in brain injury caused by ICH [6]. Tumor necrosis factor-α (TNF-α) and interleukin-1 (IL-1) are secreted by neuronal cells and activated glial cells, such as microglia and astrocytes [7, 8]. These cytokines ultimately destroy the blood–brain barrier (BBB) and cause neuronal death [9]. Inhibiting TNF-α alleviates brain edema, inflammation, and neurological impairment, but has no influence on hematoma volume [10]. Serum IL-β levels are closely associated with the seriousness of the disease and cerebral edema [11]. High IL-β levels not only cause an inflammatory response, but also lead to cerebral edema and necroptosis after ICH. Inhibiting the production of mature IL-β suppresses matrix metalloproteinase (MMP)-9 expression and activity, and reduces degradation of zonula occludens-1, which attenuates BBB leakage and brain edema after ICH [12]. Apoptosis is another type of cell death that occurs in perihematomal brain tissue after ICH. Up to now, the B-cell lymphoma 2 (Bcl-2) family protein was thought to participate in aspects of apoptotic regulation. Bcl-2 and Bcl-2-related X-protein (Bax) are important members of the Bcl-2 family; they are expressed in the central nervous system and are strongly linked to apoptosis. A previous study reported that the number of apoptotic cells in the brain gradually increases post-ICH, peaking at 72 h, accompanied by a decrease in gene and protein expression levels of Bcl-2 and increased Bax expression [13]. Treatment with dexamethasone, an anti-inflammatory corticosteroid, reduced the number of terminal deoxynucleotidyl transferase dUTP nick end labeling (TUNEL)-positive cells in the area surrounding the hematoma on day 3 in rats [14].

Peroxisome proliferator-activated receptor β/δ (PPAR-β/δ) is a ligand-activated transcription factor of the PPAR nuclear receptor family that plays important roles in the regulation of cellular metabolism by binding to sequence-specific DNA elements [15]. PPAR-β/δ plays a vital physiological role by regulating inflammation, energy homeostasis, cell proliferation, and differentiation [16, 17]. Besides its expression in peripheral organs, PPAR-β/δ is also highly expressed in the central nervous system [18]. The anti-inflammatory effects of PPAR-β/δ occur via a reduction in the binding of nuclear factor (NF)-κB to DNA by interfering with NF-κB subunit p65, thereby inhibiting the transcription of NF-κB target genes, such as inducible nitric oxide synthase (iNOS), cyclooxygenase-2 (COX-2), and TNF-α [19, 20]. PPAR-β/δ agonists play a neuroprotective role by exerting anti-inflammatory effects in neurological disorders, such as stroke and neurodegenerative disorders [20]. Treatment with GW0742, a PPAR-β/δ agonist, decreased cerebral infarction volume, BBB leakage, and the levels of proinflammatory cytokines, such as IL-1β, TNF-α, and IL-6, in patients with cerebral ischemic stroke [21]. Moreover, PPAR-β/δ promotes the survival of neurons under stress conditions by inhibiting apoptosis [22, 23]. One study showed that GW0742 decreased brain infarct volume in a middle cerebral artery occlusion (MCAO) model, partly by reducing apoptosis [24]. In PPAR-β/δ knockout mice, the cerebral ischemia-induced infarct size was increased two-fold compared to that in wild-type mice [25]. Overexpression of PPAR-β/δ alleviates neuronal apoptosis by inhibiting the NF-κB/MMP-9 pathway and relieving early brain damage in subjects with a subarachnoid hemorrhage [26]. However, whether activation of PPAR-β/δ has a neuroprotective effect against ICH-induced brain injury remains to be determined. To examine this hypothesis, the current study pretreated collagenase-induced ICH mice with GW0742. The results indicated that activation of PPAR-β/δ significantly improved brain injury, possibly by reducing inflammation and apoptosis.

## Materials and Methods

### Experimental Animals and the ICH Model

The adult male C57BL/6 mice (aged 8–10 weeks; weight, 25–30 g) used in this study were obtained from the Comparative Medical Center of Yangzhou University. All mice were housed in an air-conditioned (22 ± 1 ℃) room with a 12 h light/dark cycle and freely available water and food. All animal use and experimental protocols were approved by the Institutional Animal Care and Use Committee and the Animal Ethics Committee of Yangzhou University [SYXK (Su) IACUC 2017-0045].

To generate the ICH model, mice were anesthetized with 10% chloral hydrate (0.25 ml/100 g, intraperitoneally, i.p.) and placed in a stereotaxic frame. Bacterial collagenase type IV (0.05 U) (Sigma-Aldrich Co. St. Louis, MO, USA) in 0.2 μL of saline was injected into the right caudate putamen 3.7 mm laterally and 0.2 mm anteriorly to the bregma, at a depth of 3.8 mm, as described previously [27].

The injections took 5 min, and the needle was left in place for an additional 5 min. Bone wax was used to seal the burr hole and the scalp was sutured. During the entire surgery, the body temperature of the mice was maintained at 36.5–37.5 ℃ with a heating blanket. Sham-operated mice were treated identically, except they were injected with an equal volume of normal saline.

### Agent Administration and Experimental Protocol

The selective PPAR-β/δ agonist GW0742 was dissolved in dimethyl sulfoxide (DMSO) and then diluted in saline (final DMSO concentration, 0.1%). The mice were randomly divided into three groups: sham group, vehicle (control) group (pretreated with saline at the same concentration as for DMSO 30 min before ICH), and GW0742 group (pretreated with GW0742 3 mg/kg i.p. 30 min before ICH). The duration and dose of GW0742 were based on in-house experiments and previous studies [21, 24].

### Evaluation of Neurological Impairment

#### Forelimb Placement Test

The forelimb placement test was carried out by a blinded investigator as described previously [28, 29]. The mice (n = 9/group) were grasped by the torso, allowing the forelimbs to hang free. Each mouse was moved up and down lightly to prevent struggling. Each forelimb was tested by slightly touching the ipsilateral vibrissae on the corner edge of a countertop. Animals without cerebral hemorrhage quickly placed the forelimb on the countertop. The ICH mice that placed the forelimb contralateral to the hematoma side were considered potentially brain-damaged. All mice were tested ten times, and the percentage of mice that placed the left forelimb was calculated as the forelimb placing score.

#### Corner Turn Test

The animals (n = 9/group) were placed in an area with a 30° corner, and turned left or right to exit that area. Only turns including full rearing along either wall were recorded. According to the extent of injury, ICH mice tend to turn to the side ipsilateral to the damage [29]. The test was repeated ten times, with an interval of at least 30 s between each test, and the percentage of right turns was recorded.

#### Rotarod Test

Motor coordination was assessed with the rotarod test, as described previously [30, 31]. In brief, animals (n = 9/group) were placed on a rotating rod located 22 cm above the platform. The diameter of the rod was 3 cm and it rotated at a speed of 4–40 rpm over a 5-min period. Mice that achieved stable baseline performance during the 3-day training period were included in the experiment. Mice that fell off the testing rod were placed back on the rod with minimal disturbance. The baseline latency for the animal to fall off the rod was recorded on the final day of training. After the ICH operation, selected mice were tested three times/day on days 1 and 3, and the average latency to falling was recorded.

### Blood–Brain Barrier Permeability

Extravasation of Evans blue (EB) dye and fluorescein isothiocyanate (FITC)-dextran was performed to examine the BBB permeability on day 3, [32, 33].

#### Evans Blue Extravasation

Briefly, 4 ml/kg EB dye (E8010-1; Solarbio, Shanghai, China; 2% in saline) was injected into the tail vein of animals (n = 5/group) 3 h before they were killed. Then, the mice were perfused with 40 ml of cold saline transcardially. The cerebral tissues were rapidly harvested, weighed, and homogenized in 0.1 M phosphate-buffered saline (PBS). The samples were centrifuged at 14,000 rpm and 4 ℃ for 30 min after grinding. The same volume of 50% trichloroacetic acid was mixed with the supernatant, and the mixture was left to incubate overnight at 4 ℃. The samples were centrifuged again, and the EB dye was measured by a spectrophotometer at 610 nm and quantified according to a standard curve. The results are provided as micrograms of EB dye/grams of tissue.

#### FITC-Dextran Leakage

BBB permeability was also evaluated using 70 kDa FITC-dextran. Mice (n = 5/group) were injected with 70 kDa FITC-dextran (MS0905; MKbio, Shanghai, China) in PBS at 50 mg/kg into the tail vein. Animals were perfused with pre-chilled PBS 15 min after the dextran injection. Following perfusion, the brain tissue was removed, homogenized with 20% trichloroacetic acid, and centrifuged at 12,000 rpm for 10 min. The supernatant was isolated, and relative fluorescence was measured at excitation and emission wavelengths of 493 and 520 nm. The results were calculated using FITC-dextran as the standard, and the values are expressed as ng/g brain tissue.

### Brain Water Content

The wet/dry method was applied to assess brain water content and identify cerebral edema, as described previously [34]. In short, animals (n = 5/group) were decollated under deep anesthesia 3 days after ICH, and their brains were quickly harvested and separated into the ipsilateral and contralateral cerebral hemispheres. The wet weight of each part was weighed directly with an electric analytic balance. To obtain the dry weights, the samples were dried at 100 ℃ for 24 h. Brain water content was calculated as follows: brain water content (%) = [(wet weight − dry weight)/wet weight] × 100%.

### Nissl Staining

Nissl staining was used to quantify ICH-induced brain injury, as described previously [35]. The brains (n = 5/group) were dehydrated in a 10% sucrose solution for 1 day followed by a 30% sucrose solution for 2–3 days. Then, the brains were cut with a freezing microtome to obtain coronal sections (20 μm). Cresyl violet (C9140-1; Solarbio) was used to stain the brain sections at 37 ℃ for 15 min. Next, the brain sections were washed in distilled water, treated with 95% ethanol for 30 s, covered with 50% glycerin, and dried. A light microscope and digital camera were used to take images. To evaluate whether the number of surviving neurons differed significantly among the groups, cells in three independent microscopic fields were examined. The surviving neurons had round and pale nuclei, while the damaged neurons had shrunken and condensed nuclei.

### TUNEL Assay

Double TUNEL and neuronal nuclei (NeuN) staining was carried out to evaluate neuronal apoptosis on day 3 after ICH. Briefly, the slides were incubated with the primary antibody, rabbit anti-NeuN (1:100, GB11138, Servicebio, Wuhan, China), and then co-incubated with AlexaFluor488-conjugated secondary antibodies (goat anti-rabbit, 1:400, GB25303; Servicebio,) using an apoptosis detection kit (G1501; Servicebio) for 2 h. Similar to fluorescent double labeling, TUNEL-positive cells were imaged under the fluorescence microscope. Images were captured in three microscopic fields of the perihematomal area using Image-Pro Plus 6.0 (Media Cybernetics, Silver Spring, MD, USA). Data are presented as TUNEL-positive cells/mm^2^.

### Western Blot Analysis

Western blot was performed as described previously [36, 37]. The tissues surrounding the hematoma of the right brain hemispheres of five mice from each group were harvested on day 3 after ICH or the sham operation. The samples were weighed and homogenized in ice-cold lysis buffer, and the lysates were centrifuged at 13,000 rpm for 30 min at 4 ℃. The protein concentration of the lysates was assayed using a bicinchoninic acid (BCA) protein assay kit (P0010; Beyotime Institute of Biotechnology, Shanghai, China). An equal amount of protein (50 μg) in the sample was diluted in loading buffer, denatured, separated by 8–12% sodium dodecyl sulfate-polyacrylamide gel electrophoresis, and blotted onto a polyvinylidene difluoride membrane. The membranes were blocked for 2 h at room temperature with Tris-buffered saline Tween-20 (TBST) containing 5% nonfat milk, and incubated in a primary antibody (β-actin, AC004; ABclonal, Woburn, MA, USA; PPAR-β/δ, ab23673; IL-1β, ab9722; TNF-α, ab1793; Bcl-2, ab182858, GFAP, ab7260, Iba1, ab178847; Abcam, Cambridge, UK; Bax, AF820; R&D Systems, Minneapolis, MN, USA) overnight at 4℃. The membranes were washed with TBST (pH 7.4) and incubated with secondary antibody for 2 h at room temperature. Then, the membranes were washed with TBST buffer and detected by super enhanced chemiluminescence detection reagents. Immunoreactive bands were quantitatively analyzed with ImageJ software (NIH, Bethesda, MD, USA).

### Immunohistochemical Staining and Immunofluorescence

Mice (n = 5/group) were killed 3 days after ICH and perfused transcardially with sterile saline and 4% paraformaldehyde in 0.1 M phosphate buffer. The brains were harvested and fixed overnight in 4% paraformaldehyde, and dehydrated successively in 15, 20, and 30% sucrose for 24 h until they had sunk. Serial 20-μm-thick coronal sections were prepared using a freezing microtome through the hematoma and stored in PBS at 4 ℃. The sections in PBS were exposed to 0.25% Triton X-100 and 3% H_2_O_2_ to eliminate endogenous peroxidase activity, blocked in 5% normal goat serum, and incubated overnight at 4 ℃ with the primary antibodies anti-PPAR-β/δ (diluted 1:300; Abcam), anti-IL-1β (1:100; Abcam), anti-TNF-α (1:200; Abcam), anti-Bcl-2 (1:500; Abcam), anti-Bax (1:100; R&D Systems), followed by horseradish peroxidase (HRP) goat anti-mouse IgG (H + L) (1:200, AS003; ABclonal) or HRP goat anti-rabbit IgG (H + L) (1:200, AS014; ABclonal). Sections were developed with a DAB kit (Zymed Laboratories Inc., San Francisco, CA, USA). Hematoxylin was used for counterstaining. A digitized image of each section was obtained and analyzed using an Axioplan 2 microscope (Zeiss, Oberkochen, Germany). Four nonoverlapping sections (200 × 200 μm^2^) were randomly but systematically selected from the perihematomal areas, and cell counts were performed blindly to quantify the number of positive cells.

The sections for double immunofluorescence labeling were prepared as described previously. After rinsing with PBS containing 0.3% Triton X-100 for 1 h, the brain sections were blocked with 5% normal goat serum for 2 h at room temperature and then incubated with rabbit anti-PPAR-β/δ (diluted 1:300; Abcam), rabbit anti-NeuN (1:100, GB11138; Servicebio, Wuhan, China), mouse anti-Iba1 (1:200, GB12105; Servicebio), and mouse anti-GFAP (1:400, GB12096; Servicebio) overnight at 4 ℃. After washing with PBS, the slices were incubated with AlexaFluor488-conjugated secondary antibodies (goat anti-mouse, 1:400, GB25301; goat anti-rabbit, 1:400, GB25303; Servicebio) in a dark box for 2 h at room temperature. The brain slices were washed with PBS and mounted with cover slips after adding DAPI. The cellular localization of PPAR-β/δ was observed and photographed under a fluorescence microscope.

### Statistical Analysis

The experimental data are presented as mean ± standard error. Data from all experiments were quantified and analyzed using GraphPad Prism 7.0 software (GraphPad Software Inc., La Jolla, CA, USA). Comparisons between groups were carried out using the *t* test or analysis of variance. P-values < 0.05 were considered significant.

## Results

### The Expression of PPAR-β/δ was Mainly Observed in Neurons and the Levels Increased in the Perihematoma After ICH

**Fig. 1:**
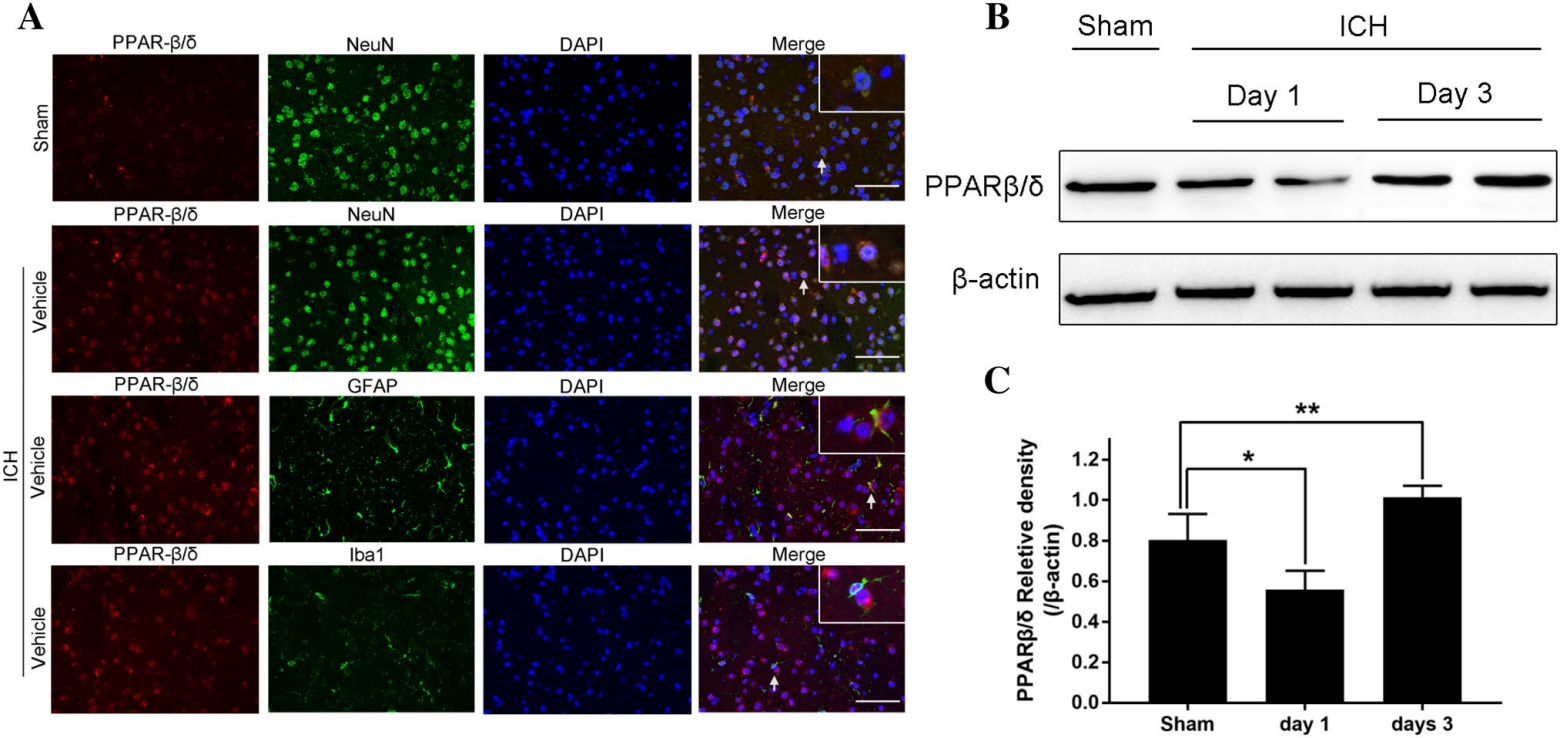
Peroxisome proliferator-activated receptor β/δ (PPAR-β/δ) levels decreased on day 1, but increased on day 3 after intracerebral hemorrhage (ICH), mainly in neurons. **a** Representative microphotographs of double immunofluorescence labeling showing that PPAR-β/δ (red) co-localized with neuronal nuclei (NeuN)-positive neurons (green) in the sham and ICH groups. **b** Western blot was used to detect the PPAR-β/δ protein level in the perihematomal area on days 1 and 3. **c** The density of the bands in the different groups is illustrated by the quantification graph. β-actin was used as the internal control. Values are mean ± SD; *P < 0.05 vs. sham group, **P < 0.05 vs. sham group (n = 5/group)

Double immunofluorescence labeling and western blotting were performed to determine the cellular localization and protein levels in perihematomal tissues after ICH. The results showed that the PPAR-β/δ colocalized with NeuN-positive neurons, but not with GFAP/Iba1-positive astrocytes/microglia, 3 days after ICH in mice. Western blotting analysis showed that the levels of PPAR-β/δ decreased significantly in the perihematomal tissue on day 1 after ICH compared to the sham-control group (P < 0.05, Fig. 1). However, the PPAR-β/δ protein level increased 3 days after ICH (P < 0.05, Fig. 1). This result indicated that ICH promoted the expression of PPAR-β/δ 3 days after ICH in mice and was mainly expressed by neurons.

### Effect of GW0742 on Neurological Deficits and Hematoma Volume After ICH

**Fig. 2:**
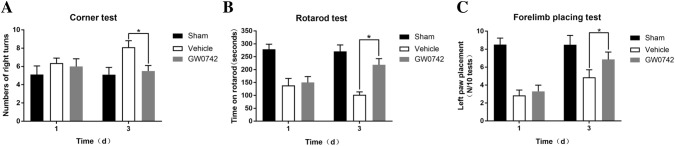
Analysis of behavioral changes on days 1 and 3. **a** The number of right turns indicated the neurological deficits that occurred on day 3 post-ICH, with recovery on day 3 occurring in response to the GW0742 treatment; **b** The time on the rotarod in the rotarod test increased significantly in response to GW0742 on day 3; **c** Left paw placement on the forelimb placing test was improved by GW0742 on day 3. Values are means ± SD; ^*^P < 0.05 vs. vehicle group (n = 9/group)

**Fig. 3:**
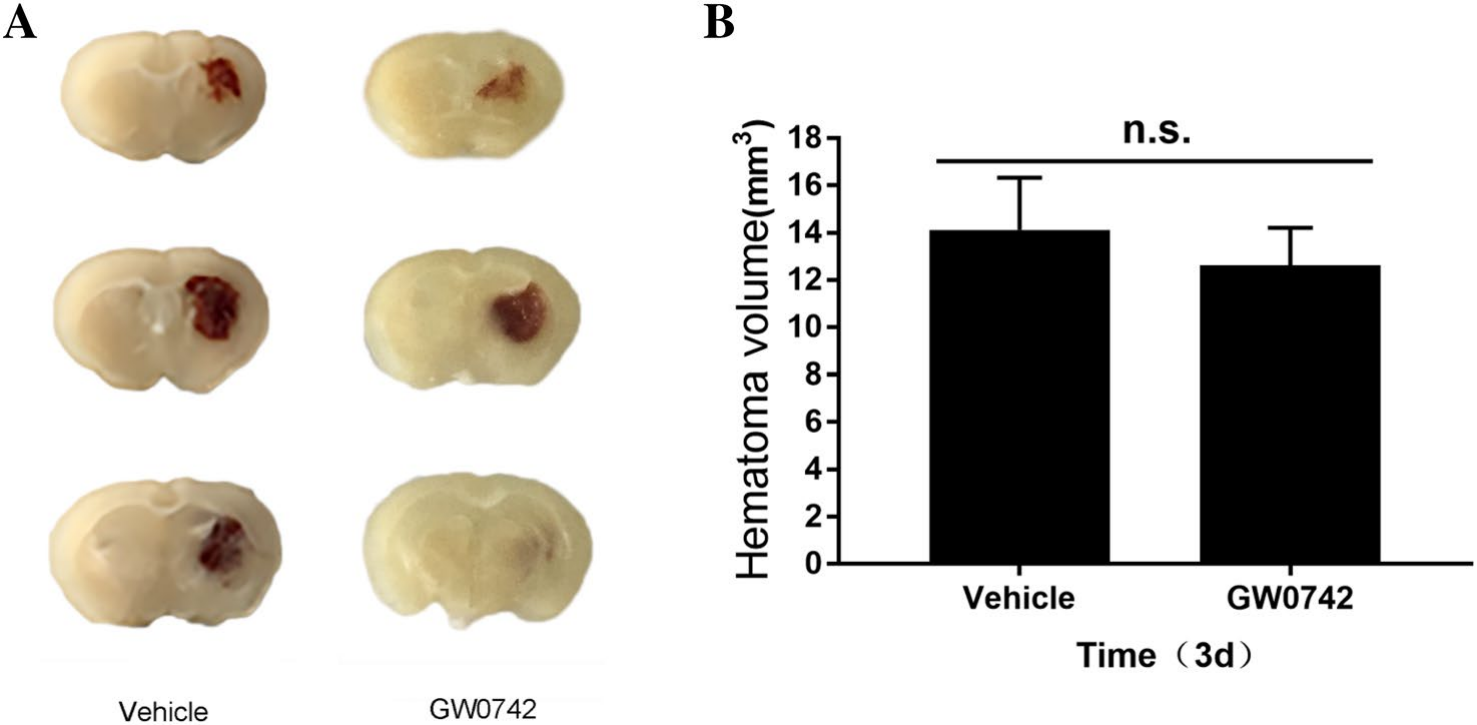
Analysis of hematoma size. **a** Hematoma volumes on day 3 were calculated based on morphometric measurements. Scale bar = 2 mm; **b** Quantification of hematoma volume. Values are mean ± SD. n.s., no significant difference (n = 5/group)

A range of behavioral tests were performed to estimate acute effects of GW0742 on sensory and motor functions on days 1 and 3 after ICH. The corner test, rotarod test, and forelimb placement test were used to evaluate nerve dysfunction in the mice. No significant difference was observed between the vehicle and GW0742 groups on day 1, but the GW0742 pretreatment significantly reduced the increase in right turns in the vehicle group on day 3 (P < 0.05, Fig. 2a). No significant differences were observed between the vehicle and GW0742 groups on the rotarod test on day 1. However, GW0742 significantly prolonged the time spent on the rotarod by the ICH mice on day 3 compared to the vehicle group (P < 0.05, Fig. 2b). The frequency of left paw placements in the GW0742 group increased significantly compared to that in the vehicle group (P < 0.05, Fig. 2c). Morphometric measurements were used to determine the effect of GW0742 on hematoma volume. The result revealed that the GW072 pretreatment did not affect hematoma size on day 3 after ICH (P > 0.05, Fig. 3).

### Effect of GW0742 on Brain Edema and Blood–Brain Barrier Permeability

**Fig. 4:**
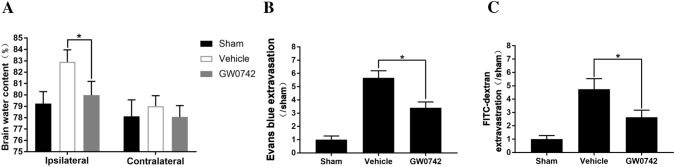
Brain edema and blood–brain barrier (BBB) damage on day 3. **a** Brain water content, **b** Evans blue extravasation. **c** Fluorescein isothiocyanate (FITC)-dextran extravasation. Values are mean ± SD; ^*^P < 0.01 vs. vehicle group (n = 5/group)

Mice subjected to ICH showed significantly increased brain water content, as well as BBB leakage in the ipsilateral hemispheres, on day 3 after the operation. As shown in Fig. 4, administering GW0742 significantly reduced brain edema on day 3 after ICH compared to the vehicle group (P < 0.01, Fig. 4a). In addition, a high level of EB dye extravasation was detected on day 3 after ICH, and GW0742 pretreatment attenuated the EB leakage compared to the vehicle group (P < 0.01, Fig. 4b). Leakage of FITC-dextran was also evaluated. As shown in Fig. 4c, a low FITC-dextran level was detected in the sham group. The level of brain FITC-dextran increased significantly 3 days post-ICH, which was reduced by administering GW0742 (P < 0.05, Fig. 4c).

### Effect of GW0742 on Neuronal Death in the Perihematoma After ICH

**Fig. 5:**
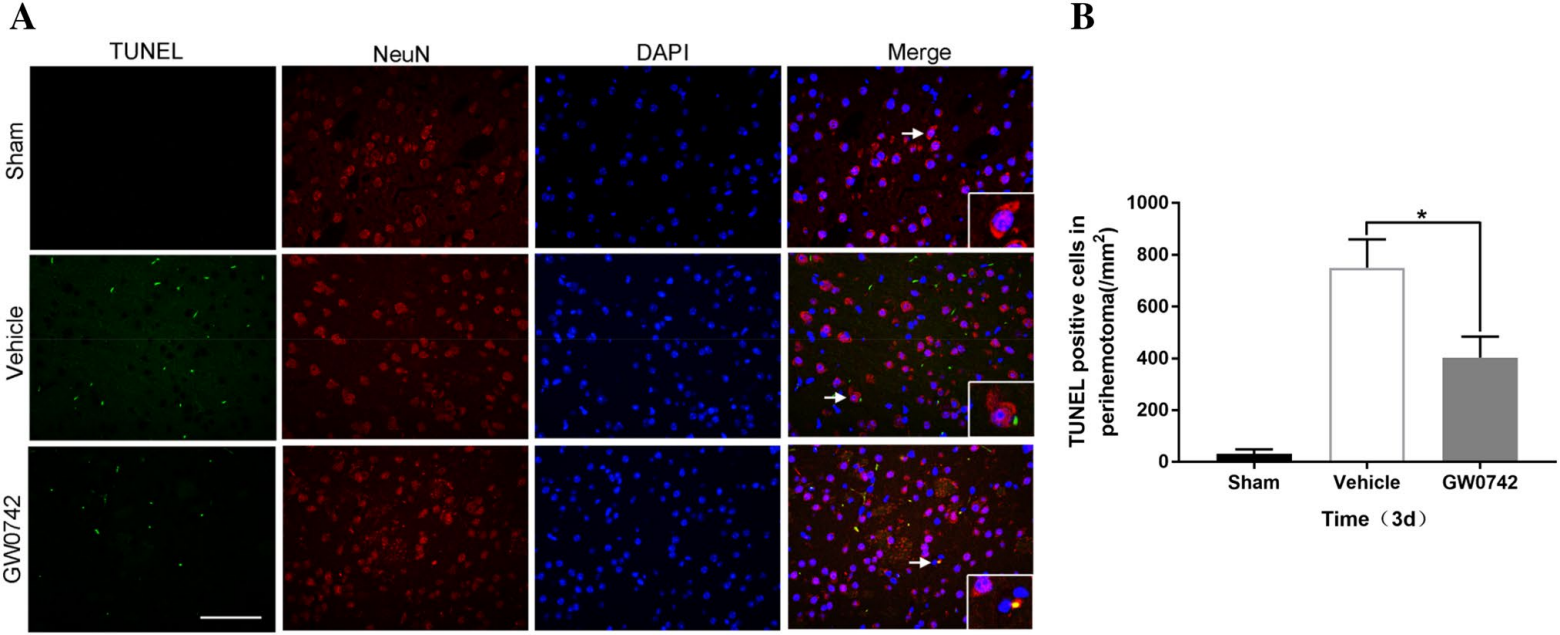
Apoptotic neuronal cell analysis. **a** Representative photographs showing double terminal deoxynucleotidyl transferase dUTP nick end labeling (TUNEL)-positive (green) and neuronal nuclei (NeuN) (red) immunostaining in the perihematomal area on day 3 (bar = 50 μm). **b** Quantification of TUNEL-positive neuronal cells. Values are mean ± SD; *P < 0.05 vs. vehicle group (n = 5/group)

**Fig. 6:**
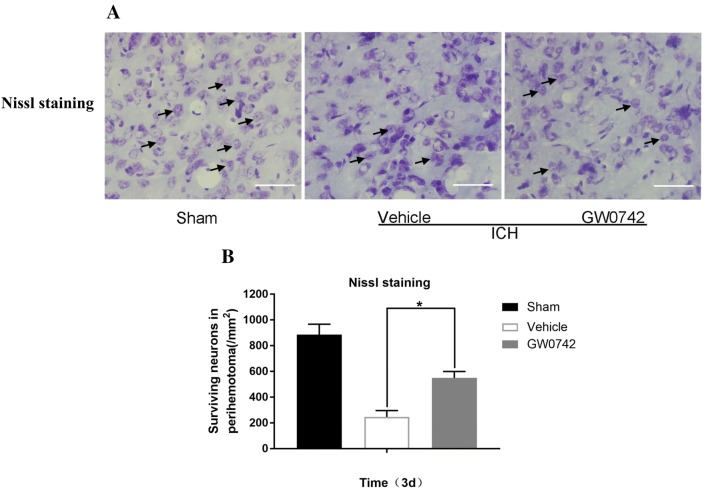
Pretreatment with GW0742 increased the number of surviving neurons in the perihematomal area after ICH. **a** Representative Nissl-stained image showing the surviving and disintegrated neurons in the perihematomal area on day 3 (bar = 50 μm). **b** Quantification of surviving neurons. Values are mean ± SD; *P < 0.05 vs. vehicle group (n = 5/group)

The neuroprotective role of the GW0742 pretreatment was assessed in mice with ICH-induced brain injury in the following ways. First, TUNEL staining was used to determine neuronal apoptosis in the perihematomal brain tissue on day 3. The TUNEL-positive neurons increased significantly in the tissue surrounding the hematoma in the ICH mice (Fig. 5a). The GW0742 pretreatment decreased the number of TUNEL-positive neurons post-ICH compared to the vehicle group (P < 0.01, Fig. 5b). Nissl staining was performed to assess the diversity of neurons that survived (Fig. 6). The number of surviving neurons decreased significantly in the ICH mice. The morphology of the dead neuronal cells included small, darkly stained, shrunken nuclei in the perihematoma on day 3 (Fig. 6a). After the GW0742 pretreatment, the number of surviving neurons increased compared to the vehicle group (P < 0.05, Fig. 6b).

### GW0742 Upregulates the PPAR-β/δ Level in the Perihematoma After ICH

**Fig. 7:**
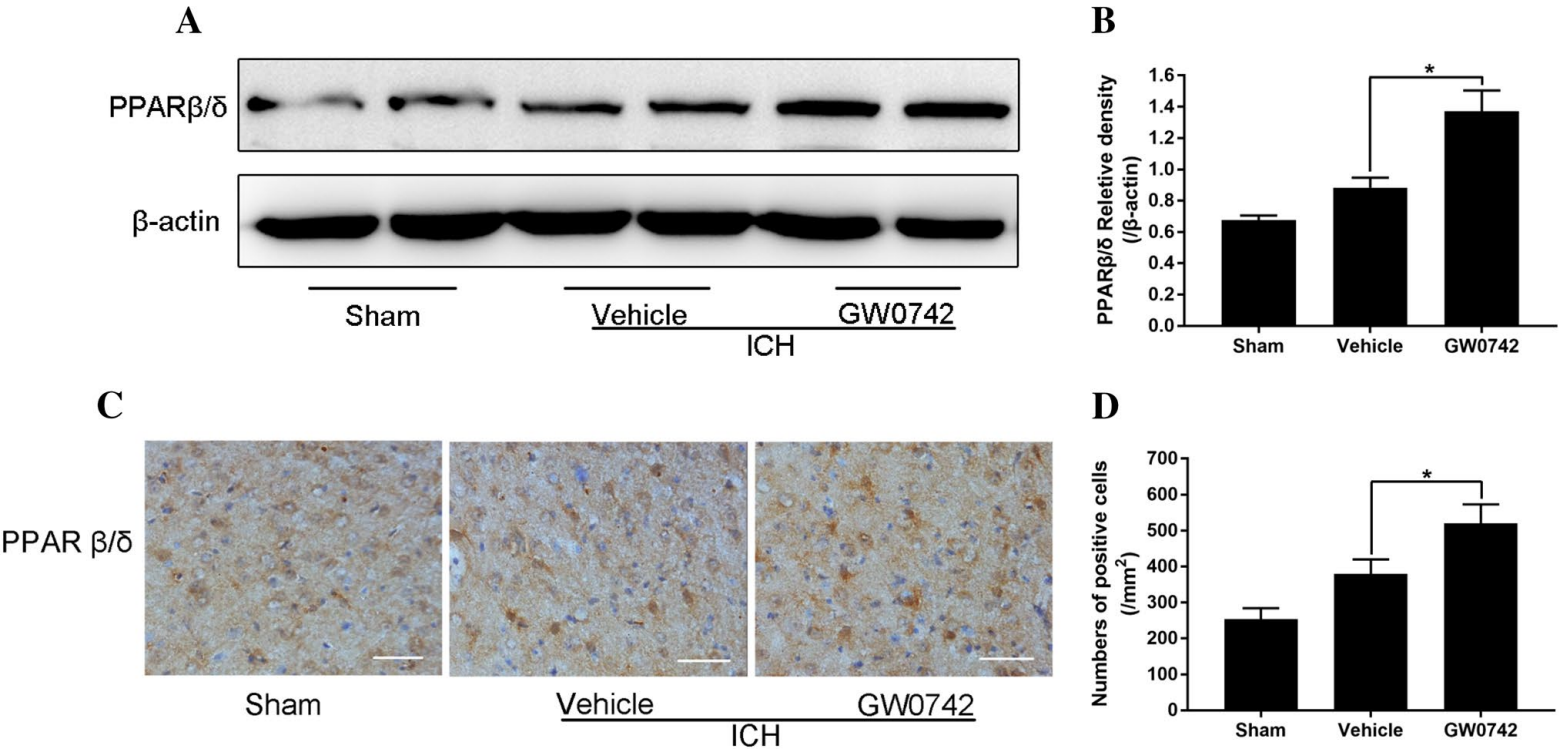
Pretreatment with GW0742 increased the PPAR-β/δ levels in ICH lesions on day 3. **a** Western blotting for PPAR-β/δ, assessed using perihematomal tissue on day 3. **b** The optical density of PPAR-β/δ relative to β-actin in the different groups is illustrated by the quantification graph. **c** Representative photographs of PPAR-β/δ immunostaining in the perihematomal area on day 3 (bar = 50 μm). **d** The positive cells shown in C were counted. Values are mean ± SD; *P < 0.05 vs. vehicle group (n = 5/group)

We explored the effect of ICH on the level of PPAR-β/δ and determined whether the GW0742 pretreatment increased the level of PPAR-β/δ in perihematomal tissue. The levels of PPAR-β/δ were verified by western blot analysis and immunohistochemical staining on day 3 after ICH. Western blotting indicated that the PPAR-β/δ level was higher after ICH, and GW0742 pretreatment further increased the PPAR-β/δ level (P < 0.05, Fig. 7a, b). Immunohistochemical staining also demonstrated that there were more PPAR-β/δ-positive cells in the GW0742 group than in the vehicle group (P < 0.05, Fig. 7c, d).

### GW0742 Attenuates the ICH-Induced Neuroinflammatory Response in the Perihematoma

**Fig. 8:**
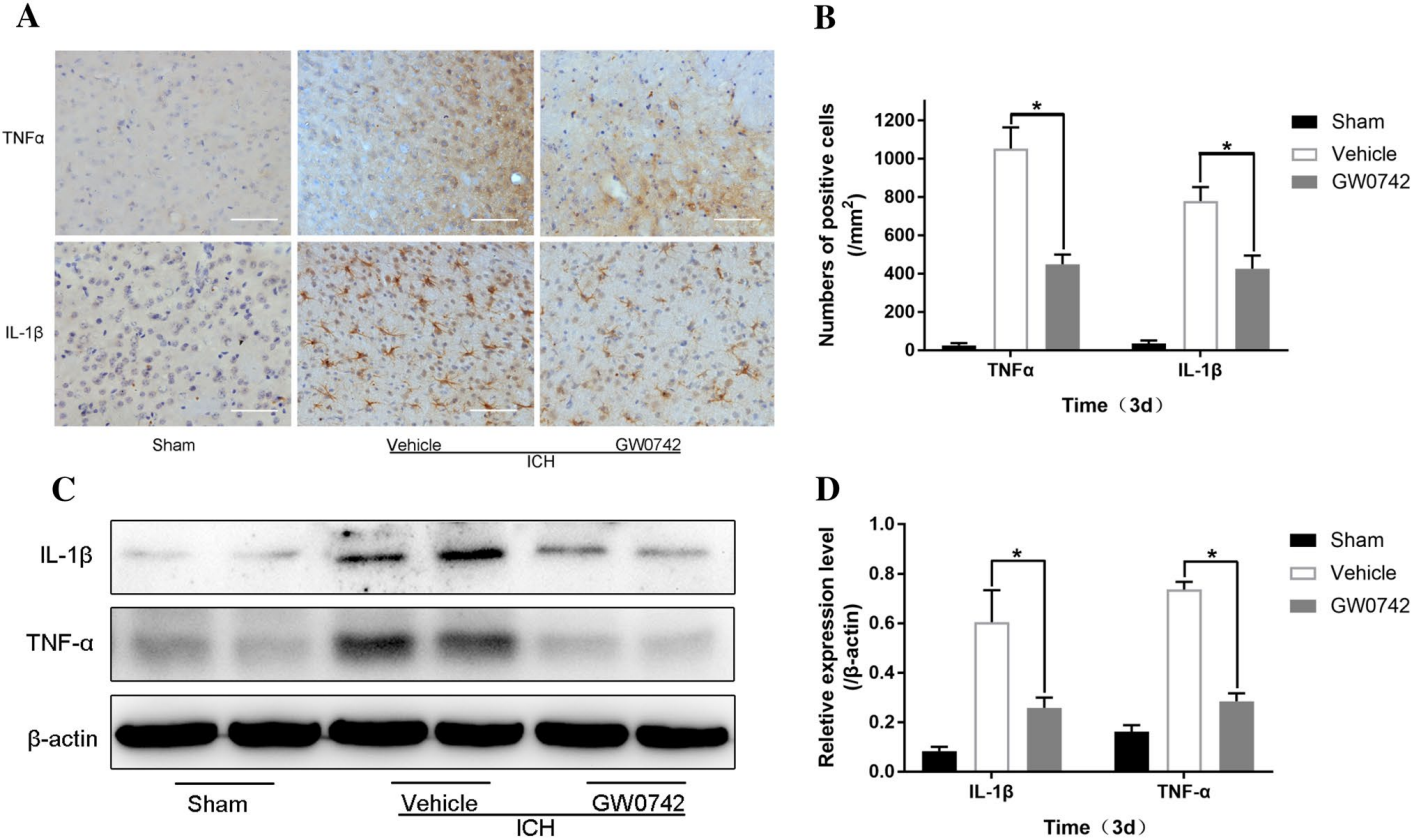
GW0742 reduced ICH-induced production of tumor necrosis factor (TNF)-α and interleukin (IL)-1β in the perihematomal area on day 3 after ICH. **a** Representative photographs of TNF-α and IL-1β immunostaining in the perihematomal tissue on day 3 after ICH (bar = 50 μm). **b** The numbers of TNF-α- and IL-1β-positive cells were counted in each group. **c** TNF-α and IL-1β expression in the perihematomal tissue was determined by western blot; β-actin was the loading control. **d** The optical density of TNF-α and IL-1β relative to β-actin in the different groups is illustrated by the quantification graph. Values are mean ± SD; *P < 0.05 vs. vehicle group (n = 5/group)

The inflammatory response exacerbates the brain damage that occurs after ICH, eventually resulting in cerebral injury, BBB dysfunction, and massive brain cell death [38]. In our experiment, the localization of TNF-α and IL-1β proteins was identified by immunohistochemistry on day 3 after ICH. The levels of TNF-α and IL-1β in perihematomal tissue are shown in Fig. 8a. Strong positive TNF-α and IL-1β staining was observed in the perihematomal area after ICH. Administering GW0742 significantly decreased the number of positive cells expressing TNF-α and IL-1β on day 3 after ICH compared to the vehicle group (P < 0.05, Fig. 8b).

Western blotting was used to further investigate the expression levels of TNF-α and IL-1β. Consistent with the immunohistochemistry results, ICH induced a significant increase in TNF-α and IL-1β protein levels (Fig. 8c). GW0742 significantly reduced the levels of TNF-α and IL-1β compared to the vehicle group (P < 0.05, Fig. 8d).

### GW0742 Mitigates the ICH-Triggered Apoptotic Response in the Perihematoma

**Fig. 9:**
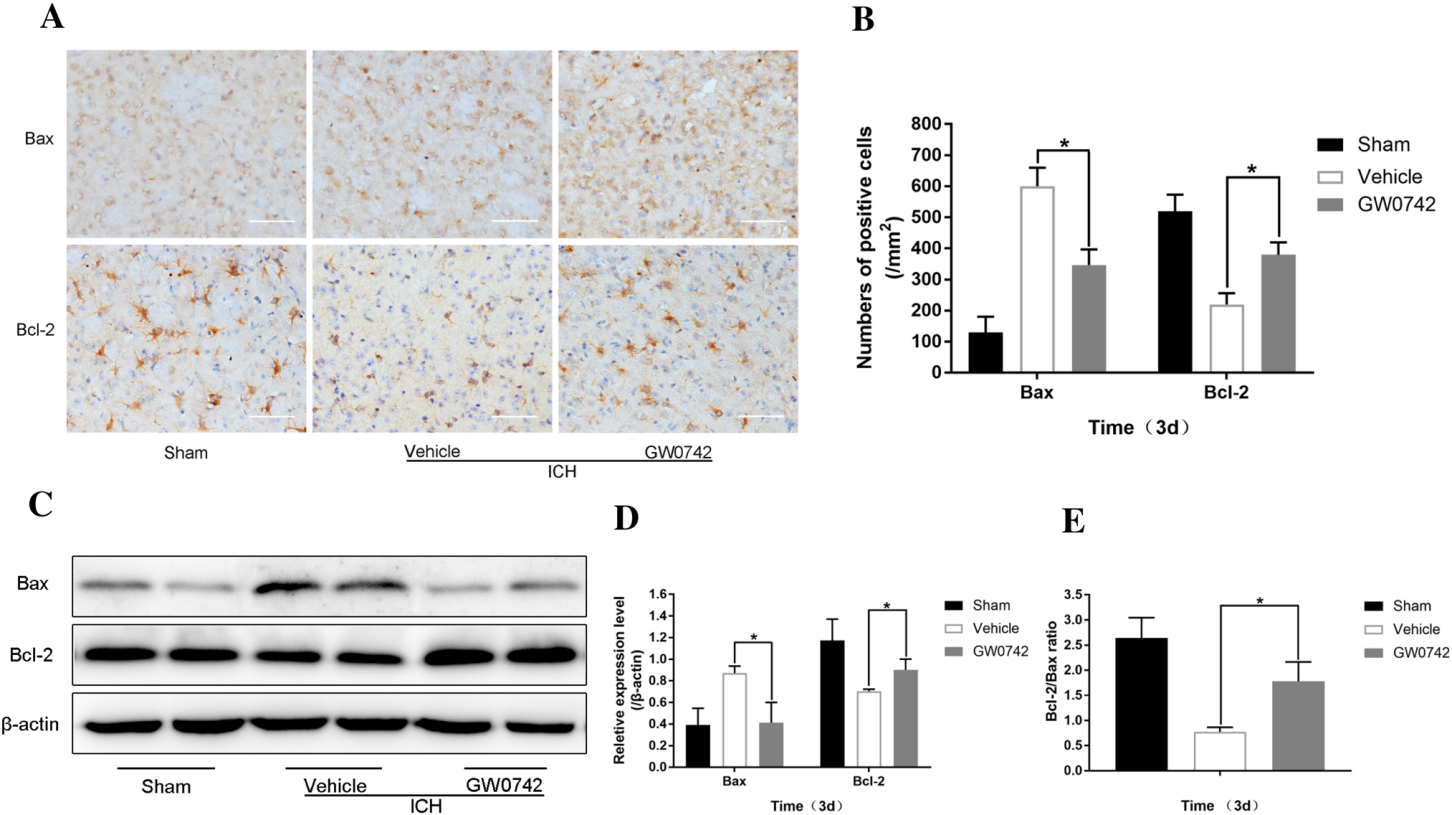
GW0742 decreased Bcl-2-related X-protein (Bax) levels and increased B-cell lymphoma 2 (Bcl-2) levels and the Bcl-2/Bax ratio in the perihematomal area on day 3 after ICH. **a** Representative photographs of Bax and Bcl-2 immunostaining in the perihematomal tissue on day 3 (bar = 50 μm). **b** The numbers of Bax and Bcl-2-positive cells were counted. **c** The relative protein levels were determined by western blot; β-actin was the loading control. **d**, **e** The optical density of Bax and Bcl-2 relative to β-actin, and the Bcl-2/Bax ratio in the different groups are illustrated by the quantification graph. Values are mean ± SD; *P < 0.01 vs. vehicle group in all graphs (n = 5/group)

Previous studies have shown that activation of apoptosis plays an important role in the pathology of brain injury triggered by ICH [39, 40]. The results of TUNEL staining (Fig. 5) demonstrated that GW0742 strongly inhibited ICH-induced apoptosis. To further study the anti-apoptotic effect of GW0742 in ICH-induced brain injury, the Bax and Bcl-2 proteins were localized by immunohistochemistry on day 3 after ICH (Fig. 9a). A significant increase in the number of Bax-positive cells was observed in the surrounding hematoma tissue of the ICH mice. After administering GW0742, Bax immunoreactivity decreased significantly in the perihematomal tissue (Fig. 9b). The number of Bcl-2-positive cells decreased following ICH, which was significantly reversed by the GW0742 pretreatment (P < 0.01, Fig. 9b).

The current study also used western blotting to detect Bax and Bcl-2 levels (Fig. 9c). Similar to the immunohistochemistry results, the Bax level decreased in the GW0742 group after ICH. In addition, the Bcl-2 level increased in the GW0742 group (P < 0.01, Fig. 9d). Therefore, the Bcl-2/Bax ratio increased after the GW0742 pretreatment on day 3 after ICH (P < 0.01, Fig. 9e).

### GW0742 Inhibits Microgliosis and Astrogliosis in the Perihematoma After ICH

**Fig. 10:**
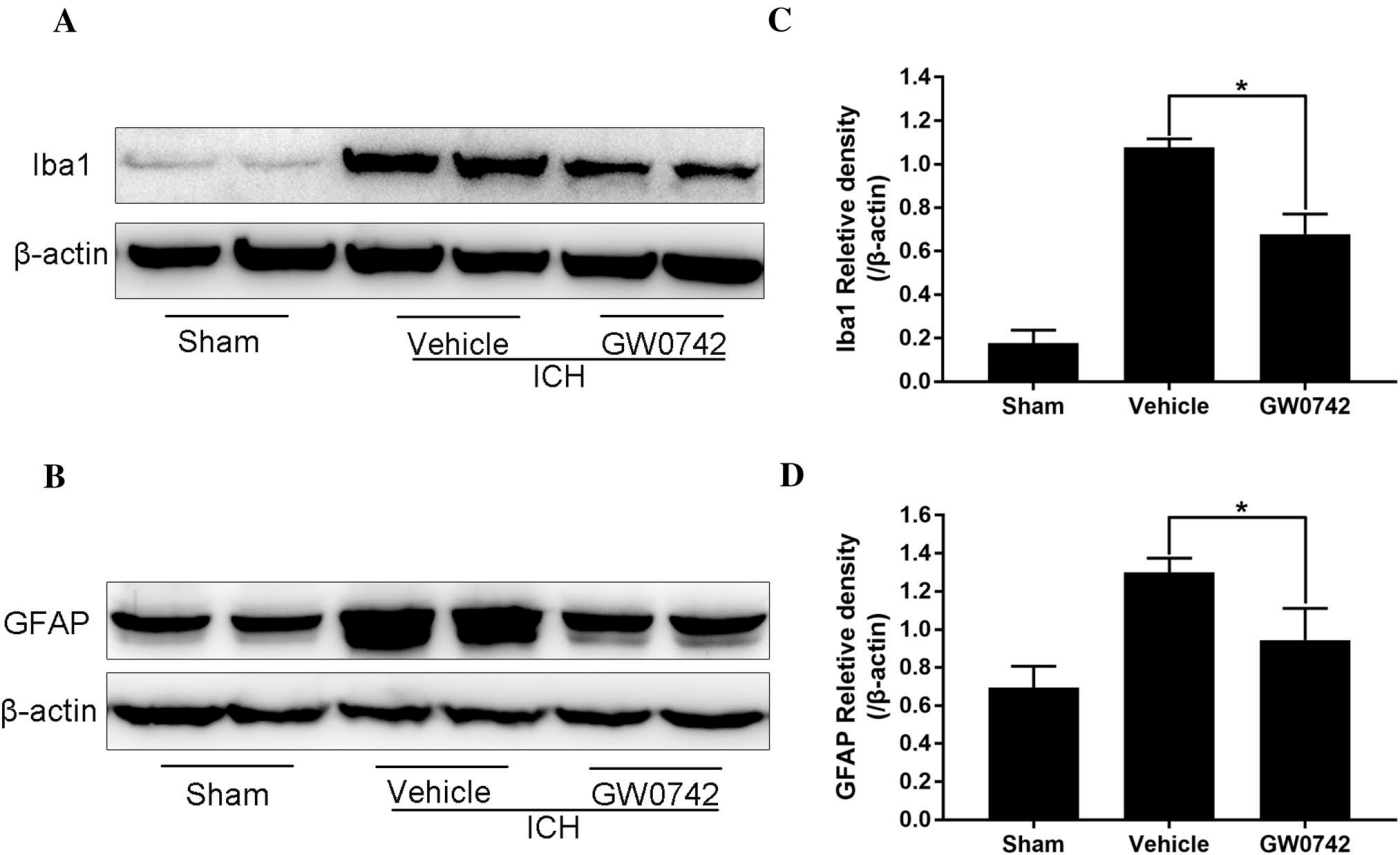
Pretreatment with GW0742 inhibited the activation of microglia and astrocytes in the perihematoma on day 3 following ICH. **a**, **b** Western blotting for Iba1 and GFAP as assessed in the perihematomal tissue on day 3. **c**, **d** The optical density of Iba1 and GFAP relative to β-actin in the different groups is illustrated by the quantification graph. Values are mean ± SD; *P < 0.05 vs. vehicle group (n = 5/group)

Emerging evidence suggests that activation of microglia and astrocytes contributes to brain injury after ICH. It may be possible to alleviate ICH-induced tissue damage by modulating the inflammatory processes mediated by microglia and astrocytes [41]. A western blot of Iba1 (a microglia marker) and GFAP (an astrocyte marker) was performed to further elucidate the effects of PPAR-β/δ activation by GW0742 on microgliosis and astrogliosis in perihematomal tissue on day 3 after ICH. As shown in Fig. 10a, the Iba1 protein level increased significantly on day 3 after ICH. GW0742 significantly decreased the Iba1 level compared to the vehicle group (P < 0.05, Fig. 10c). In addition, GW0742 reduced the level of GFAP in the perihematomal tissue after ICH (P < 0.05, Fig. 10b, d).

## Discussion

Here, we report that activation of PPAR-β/δ by GW0742 in an ICH model alleviated brain injury by reducing inflammation and apoptosis. Importantly, pretreatment with the PPAR-β/δ agonist conferred remarkable neuroprotection by reducing ICH-induced neuronal cell loss. Neuronal death is a crucial pathological characteristic of ICH. Loss of neurons is strongly linked to the poor outcome of patients with ICH [40]. Many factors that activate post-ICH pathophysiological pathways cause cell death in perihematomal tissue. The current research links PPAR-β/δ-mediated attenuation in brain injury with the conservation of neurons in a collagenase-induced mouse model of ICH. The current study showed that the neuroprotective effect of PPAR-β/δ activation may not have occurred through any reduction in the hematoma volume. In the present study, the number of surviving neurons in the perihematoma decreased, and the number of apoptotic neuronal cells increased, after ICH, which was attenuated by the GW0742 pretreatment. These results suggest that activating PPAR-β/δ mitigated brain injury in experimental ICH mice.

In our study, the GW0742 pretreatment decreased the production of cytokines, microgliosis, and astrogliosis in perihematomal tissue after ICH. This finding strongly supports the hypothesis that PPAR-β/δ activation attenuates the expression of pro-inflammatory cytokines; this mitigates disruption of the BBB and decreases brain water content simultaneously [21, 24]. Many studies have shown that IL-1β and TNF-α contribute to the pathogenesis of, and exacerbate, Alzheimer’s disease, spinal cord injury, and MCAO-induced brain damage [42–44]. Increasing evidence indicates that iNOS, COX-2, and various chemokines increase in response to activated IL-1β and TNF-α, leading to the inflammatory response as well as brain damage [45]. Numerous cytokines increase in the ICH brain, where many of those, such as IL-1β and TNF-α, play a role in brain injury [8]. It was demonstrated in a model of spinal cord injury that the progression of tissue injury is regulated by IL-1β and TNF-α [46]. However, GW0742 reduced the expression of IL-1β and TNF-α in spinal cord tissue from spinal cord injured mice [47]. TNF-α is involved in the damage to the BBB that leads to brain edema [48]. Previous studies have suggested that suppressing TNF-α alleviates the inflammatory process [49]. Anti-TNF-α with an infliximab analog attenuated neurological impairment in an ICH model by reducing the brain edema caused by neuroinflammation, indicating that TNF-α is involved in the pathological progress of brain damage after ICH [10, 50]. Di Paola et al. reported that GW0742 pretreatment reduced acute inflammation by attenuating IL-1β and TNF-α production in an acute lung injury model [51]. Our data showed that microgliosis and astrogliosis were aggravated, and the levels of IL-1β and TNF-α increased, on day 3 after ICH. However, GW0742 pretreatment decreased the levels of GFAP, Iba1, and cytokines, accompanied by attenuation of BBB damage and brain water content. The GW0742 treatment alleviated ICH-induced neurofunctional deficits compared to the vehicle group. Furthermore, the GW0742 treatment significantly improved behavioral impairments, as evidenced by a decrease in the number of right turns during the corner test and increased time spent on the rotarod, and by an increase in the number of left paw placements during the forelimb placement test. The present data indicate that GW0742 activates PPAR-β/δ, which suppresses the neuroinflammatory response after ICH, supporting the idea that PPAR-β/δ has a significant regulatory effect on inflammation-induced brain injury.

Mounting evidence indicates that PPAR-β/δ is involved in the antiapoptotic process in many neurological diseases, such as subarachnoid hemorrhage, MCAO, and Alzheimer’s disease [26]. It is clear that apoptosis of neurons plays a crucial role in stroke-induced brain injury. Thrombin, blood components, and the induction of inflammation stimulate central factors leading to neuronal apoptosis after ICH [52]. The apoptosis pathways have been classified into extrinsic and intrinsic apoptotic pathways. The extrinsic apoptotic pathway requires cell surface receptors, whereas the intrinsic pathway is initiated by mitochondrial outer membrane permeabilization and the Bcl-2 family. Bax is a member of the Bcl-2 family that promotes neuronal apoptosis; the other member is the anti-apoptotic protein Bcl-2 [40]. The Bax-Bax heterodimer forms with increased Bax expression, resulting in apoptosis. In contrast, the formation of the Bcl-2-Bax heterodimer results from the expression of Bcl-2 increases, which leads to anti-apoptosis. The Bcl-2/Bax ratio indicates the likelihood of cell apoptosis or survival. Cell survival following brain injury can be improved by increasing the Bcl-2/Bax ratio [53]. The importance of apoptosis in brains injured by ICH has been demonstrated [54]. Peak expression of apoptosis is observed on day 1 and lasts up to 3 days after ICH. Most of the apoptotic cells are neurons surrounding the hematoma [55]. Inhibition of the apoptosis-related protein Bax or activation of the anti-apoptotic protein Bcl-2 occurs in experimental ICH [52]. In an MCAO model, GW0742 promoted the expression of Bcl-2 and reduced the expression of Bax and apoptotic cells [24]. An intrahippocampal infusion of GW0742 ameliorated Aβ_1-42_-induced apoptosis in the hippocampus by increasing the Bcl-2/Bax ratio [14]. According to our data, activating PPAR-β/δ with GW0742 significantly relieved the ICH-induced apoptotic response, as indicated by lowered expression of Bax and increased expression of Bcl-2 in the perihematomal tissue on day 3 post-ICH. The number of surviving neurons decreased significantly after ICH, which was improved by the GW0742 treatment. Furthermore, GW0742 reduced the number of TUNEL-positive neurons in the perihematoma after ICH. Thus, this study demonstrated that ICH-induced neuronal apoptosis was decreased though the activation of PPAR-β/δ by GW0742.

We utilized a collagenase-induced ICH model to explore the changes in PPAR-β/δ protein expression in the different groups. The perihematomal tissue from sham-operated mice was used to examine the expression of PPAR-β/δ, which was downregulated significantly on 1 day but upregulated on day 3. PPAR-β/δ expression increased in response to GW0742 on day 3 in the ICH mice. Death of neurons in the perihematoma tissue could have led to the decrease of PPAR-β/δ seen on day 1 after ICH, as it is highly expressed in cells in the caudate putamen area of the mouse brain [56, 57]. The acute inflammatory response induced by ICH may have triggered negative feedback to downregulate the expression of PPAR-β/δ. The decrease in PPAR-β/δ is in agreement with results pertaining to experimental spinal cord injury, MCAO, and subarachnoid hemorrhage [21, 26, 47]. However, PPAR-β/δ expression increased in the surviving neurons of the perihematomal tissue 3 days after ICH, as indicated by immunohistochemistry. A study using an MCAO model showed that PPAR-β/δ gene expression increased significantly in the ischemic hemispheres on days 1, 4, and 7 [25]. In addition, our results showed that pretreatment with GW0742 did not significantly decrease hematoma volume 3 days after ICH. It is clear that PPARs should be considered as part of the regulatory network, rather than as single receptors. PPARγ expression can be increased by activating PPAR-β/δ or PPARγ [58]. Preclinical and experimental studies have shown that PPARγ activators are capable of promoting endogenous hematoma clearance [59]. However, it is difficult to determine whether GW0742-activated PPAR-β/δ accelerated resolution of the hematoma in the delayed stage of ICH. Thus, future studies are needed to explore the effect of GW0742 pretreatment on hematoma clearance in the later stages of ICH.

In conclusion, the current study demonstrated that activating PPAR-β/δ with GW0742 significantly prevented ICH-induced brain injury and improved neurological impairment through anti-inflammatory and anti-apoptosis mechanisms. These data enhance our understanding of the role of PPAR-β/δ in the pathophysiology of neurological damage after ICH, and indicate that activation of PPAR-β/δ may be a potential target for ICH therapy.
